# The Contribution of Noradrenergic Activity to Anxiety‐Induced Freezing of Gait

**DOI:** 10.1002/mds.28999

**Published:** 2022-04-05

**Authors:** Natasha L. Taylor, Gabriel Wainstein, Dione Quek, Simon J.G. Lewis, James M. Shine, Kaylena A. Ehgoetz Martens

**Affiliations:** ^1^ ForeFront PD Research Clinic, Brain and Mind Centre, School of Medical Sciences The University of Sydney Camperdown New South Wales Australia; ^2^ Centre for Complex Systems The University of Sydney Camperdown New South Wales Australia; ^3^ Department of Kinesiology and Health Sciences University of Waterloo Waterloo Ontario Canada

**Keywords:** Parkinson's disease, freezing of gait, anxiety, task‐fMRI, noradrenaline

## Abstract

**Background:**

Freezing of gait is a complex paroxysmal phenomenon that is associated with a variety of sensorimotor, cognitive and affective deficits, and significantly impacts quality of life in patients with Parkinson's disease (PD). Despite a growing body of evidence that suggests anxiety may be a crucial contributor to freezing of gait, no research study to date has investigated neural underpinnings of anxiety‐induced freezing of gait.

**Objective:**

Here, we aimed to investigate how anxiety‐inducing contexts might “set the stage for freezing,” through the ascending arousal system, by examining an anxiety‐inducing virtual reality gait paradigm inside functional magnetic resonance imaging (fMRI).

**Methods:**

We used a virtual reality gait paradigm that has been validated to elicit anxiety by having participants navigate a virtual plank, while simultaneously collecting task‐based fMRI from individuals with idiopathic PD with confirmed freezing of gait.

**Results:**

First, we established that the threatening condition provoked more freezing when compared to the non‐threatening condition. By using a dynamic connectivity analysis, we identified patterns of increased “cross‐talk” within and between motor, limbic, and cognitive networks in the threatening conditions. We established that the threatening condition was associated with heightened network integration. We confirmed the sympathetic nature of this phenomenon by demonstrating an increase in pupil dilation during the anxiety‐inducing condition of the virtual reality gait paradigm in a secondary experiment.

**Conclusions:**

In conclusion, our findings represent a neurobiological mechanistic pathway through which heightened sympathetic arousal related to anxiety could foster increased “cross‐talk” between distributed cortical networks that ultimately manifest as paroxysmal episodes of freezing of gait. © 2022 The Authors. *Movement Disorders* published by Wiley Periodicals LLC on behalf of International Parkinson and Movement Disorder Society

Anxiety has recently been recognized as a crucial trigger for freezing of gait (FOG).^1^, ^2^ Emotional disturbances, such as panic attacks, have been reported to occur both before and during FOG.^3^ Physiological markers of anxiety (such as elevated heart rate and skin conductance)^4^ have been found to increase before a FOG episode.^5^, ^6^ Moreover, when anxiety is induced using an immersive virtual reality (VR) plank task, patients with FOG demonstrate more frequent and more severe freezing episodes^1^, ^2^ when navigating an elevated plank compared to a plank on the ground.^2^ Despite these behavioral observations, it remains speculative as to the neural mechanisms that underpin the relationship between anxiety and FOG. Although the neural mechanisms of anxiety in PD are not well understood, anxiety has been linked to network‐level abnormalities^7^ that are consistent with an over‐engagement of the arousal system.^8^ However, a deeper understanding of how these network‐level abnormalities relate to alterations in inter‐limb coordination and freezing is needed.

One model that incorporates the multiple features of FOG is the “cross‐talk” model,^9^ which proposes that freezing arise because of inappropriate cross‐talk between competing, yet complementary pathways that are typically involved in cognitive, motor and limbic processing.^10^ This cross‐talk leads to impaired communication with the dopaminergically‐depleted basal ganglia, which leads to a paroxysmal increase in pallidal inhibitory output to brainstem gait structures, ultimately manifesting as FOG.^9^ There is substantial support for the cross‐talk model from a range of neuroimaging studies.^11^, ^12^, ^13^, ^14^, ^15^, ^16^, ^17^, ^18^, ^19^ In addition, a task‐based functional magnetic resonance imaging (fMRI) study showed limbic input is driving a processing overload in the basal ganglia during FOG.^14^ These studies suggest that FOG could occur because of dynamic dysfunctional cross‐talk across typically coordinated neural networks.^18^, ^19^ Yet, the underlying neural mechanism that shifts the brain into a vulnerable state, which results in interference from dysfunctional cross‐talk during freezing, is not well delineated.

Although individuals with Parkinson's disease (PD) are well‐known to have dysfunctional dopaminergic systems, and dopamine is often thought to promote effective systems‐level functioning in the brain,^19^ the administration of dopaminergic medication only partially ameliorate FOG,^20^ suggesting a prominent role for non‐dopaminergic pathophysiology. There is ample evidence for pathology within the ascending arousal system in individuals with PD,^21^ which are intimately linked to threat response and anxiety. A promising candidate is the locus coeruleus (LC), which is the major hub of noradrenergic neurons in the central nervous system.^22^ The LC is known to coordinate arousal and autonomic homeostasis, leading to elevated noradrenaline release at targets widely distributed throughout the cortex.^23^ In addition to these tonic effects, the downstream phasic effects of noradrenaline have been linked to increases in network‐level integration,^8^, ^24^ which in turn have been shown to facilitate the dynamic cortical interactions (ie, cross‐talk) required for higher‐order cognitive functions such as working memory.^24^

In contrast to these beneficial cognitive effects, heightened tonic LC activity has been implicated in the stress response, via its connections within the broader sympathetic circuit, which together produce acute changes in the physiology and responsiveness of neurotransmission.^25^, ^26^ Through these connections, the LC have been proposed to play a prominent role in the pathophysiology of anxiety.^27^, ^28^ Although the recruitment of the ascending noradrenergic system is critical for the normal response to threats, it has been suggested that overactivity of the LC is related to maladaptive threat responses and prolonged anxiety.^28^ Furthermore, the LC has been implicated in the exacerbation of other PD symptoms.^29^, ^30^ For example, stress‐related activation of the noradrenergic arousal system has been involved in the manifestation of tremor.^31^ In keeping with this notion, FOG has been linked to a number of measures that indicate heightened sympathetic arousal,^1^, ^6^ suggesting a novel mechanistic explanation for how anxiety “sets the stage” for FOG to occur by shifting the brain into a state that is more susceptible to cross‐talk interference. These lines of evidence give rise to the hypothesis that LC overactivity could lead to heightened anxiety in individuals with FOG. An increase in noradrenaline secondary to anxiety would raise the response gain of cortical regions across the network,^8^ essentially facilitating cross‐talk between the motor, cognitive, and limbic networks. This could ultimately result in an inability to resolve the conflict and engage resources for motor control.^10^, ^32^

It is inherently challenging to non‐invasively measure LC activity because the nucleus is small, elongated, and embedded deep in the brainstem. All of these factors make it difficult to isolate the LC with traditional neuroimaging methods.^33^ Fortunately, non‐invasive pupillometry measurements can be used as a surrogate measure of the ascending noradrenergic system.^34^ Increases in pupil diameter relate to escalations in neuronal spiking activity within the LC.^35^ Furthermore, we can infer the effects of noradrenaline on systems‐level dynamics through graph theoretical approaches, which provide a sensitive means for summarizing systems level features of brain network activity within a robust mathematical framework.^36^ For instance, the notion of cross‐talk can be operationalized by first clustering functional connectivity matrices into tight‐knit communities, and then estimating the extent to which individual regions “participate” in multiple communities—this participation coefficient value should be elevated with heightened cross‐talk before a freezing event.

To date, no neuroimaging studies have used fMRI to interrogate the dynamic fluctuations in network‐level connectivity, during anxiety‐inducing contexts that can predispose individuals to freeze. Testing these ideas empirically has remained fundamentally challenging, due in part to the difficulty associated with manipulating “affective load” in combination with whole brain imaging of an immobile patient. To remedy this issue, we used a previously validated anxiety‐inducing VR gait task^2^—itself a novel extension of previous virtual‐reality gait tasks^1^, ^11^, ^15^, ^16^, ^37^, ^38^—in which individuals navigated a series of corridors (Fig. 1A) using foot pedals while simultaneous BOLD (Blood Oxygen‐Level Dependent) data was recorded. Anxiety was manipulated in the task using a set of virtual “planks” (Fig. 1B), which has been shown to induce a feeling of worry in individuals with PD.^2^ We were explicitly interested in the neural mechanism that sets the stage for an individual to experience more freezing per se, which we have published elsewhere.^11^, ^12^, ^13^, ^14^ A subset of the same participants also performed this task outside of the MRI scanner so that we could obtain pupillometry, which we used as a proxy measure of the ascending noradrenergic response. Using these multi‐modal data, we set out to test the hypothesis that anxiety‐inducing contexts lead to heightened noradrenergic tone, which then “sets the stage for freezing”^32^ by causing an increase in pathological cross‐talk across motor, cognitive, and limbic networks.

**FIG 1:**
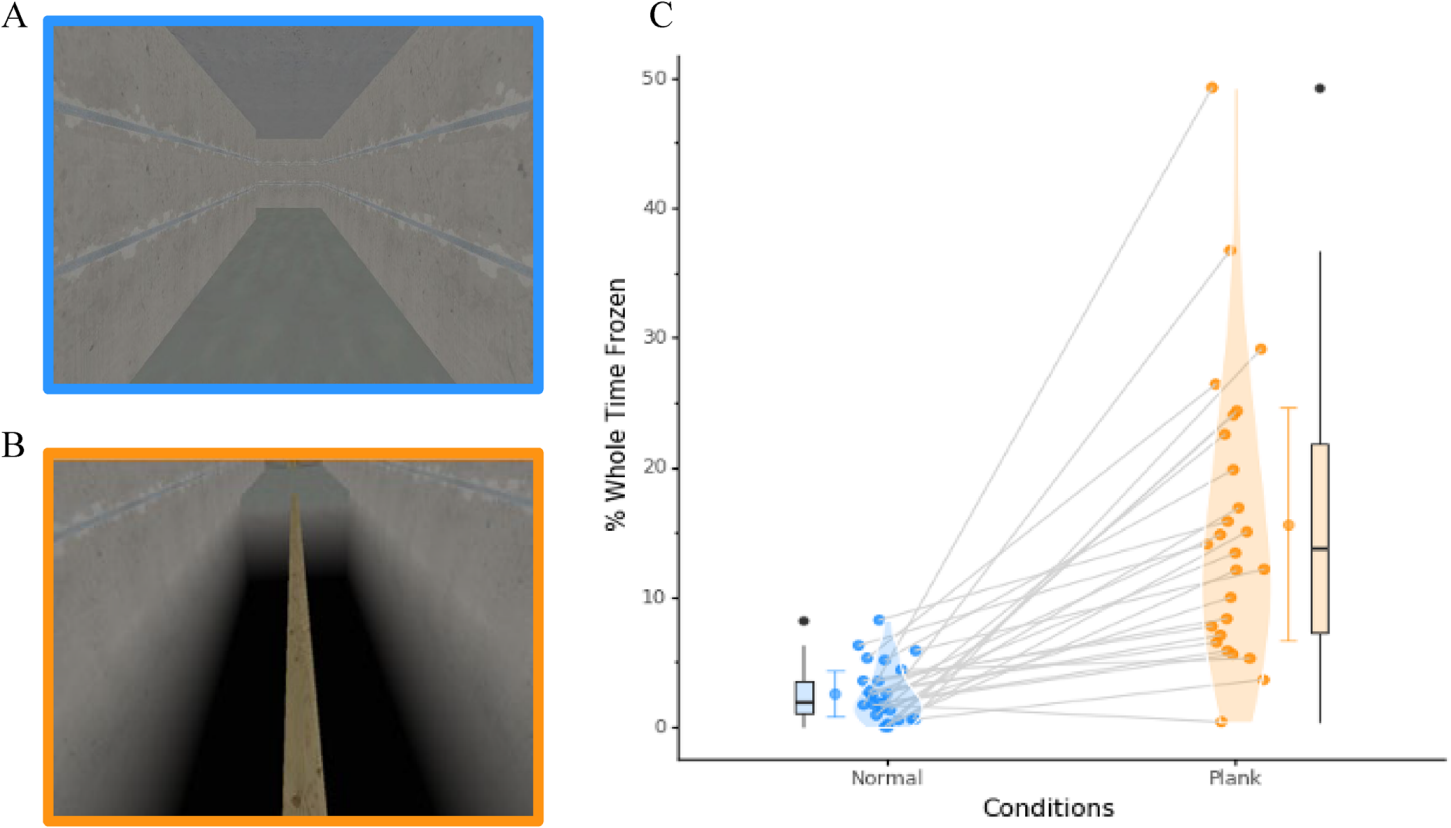
Freezing of gait increases with threatening plank condition. (**A**) Screenshot of non‐threatening (normal) condition in VR paradigm (blue); (**B**) screenshot of threatening (plank) condition in VR paradigm (orange). (**C**) Raincloud plot of each participant's average percentage of time spent frozen for both conditions, lines represent paired relationship, black dots represent outliers and barred lines represent standard deviation, with Cohen's *d* effect size = 1.63. Lines represent the paired relationship between the participants's normal and plank conditions. Violin‐plot represents the spread of the data, with the average value for both conditions represented as the point and the standard deviation as the barred lines. [Color figure can be viewed at wileyonlinelibrary.com]

## Materials and Methods

### Participants

Twenty‐nine participants with idiopathic PD and FOG participated in this study at the Brain and Mind Centre, University of Sydney. The inclusion criteria were: (1) clinical diagnosis of PD, which was confirmed using the Movement Disorder Society‐Sponsored Revision of the Unified Parkinson's Disease Rating Scale (MDS‐UPDRS); (2) score of ≥1 on question 3 of the Freezing of Gait Questionnaire; (3) clinically observed freezing, confirmed by neurologist (S.J.G.L); and (4) completion of virtual‐gait paradigm in MRI scanner during *off* state (off Parkinson's medication for 12–24 hours prior). Exclusion criteria were: (1) participants with any identified pathological abnormalities from radiologist's assessment; (2) any participants with additional neurological comorbidities. The study received ethical approval from the University of Sydney Human Research Ethics Committee. All participants provided written informed consent.

### Virtual Reality Gait Task

Participants lay supine inside the MRI scanner with a mirror mounted to the head coil for participants to see projections of VR gait task. The alternative depression of the foot pedals allowed the participant to maneuver forward through the virtual environment and encoded binary inputs corresponding to left and right footsteps were recorded on the computer. The virtual environment consisted of a series of corridors (first‐person view), that after turning a 90^o^ corner would reveal either a normal corridor or a corridor with a plank to cross. The plank condition contained two types; a narrow and a wide plank.^2^ Following previous work,^2^ freezing events in the virtual task were defined as any footstep latency that was longer than twice the mean footstep latency. Footstep latency was calculated as the time (seconds) between two subsequent footsteps.^37^, ^38^A freezing of gait episode was determined as the step before the defined freezing event, ending at one step after the defined freezing event (further details Supplementary *).

## Behavioral Analysis of the Virtual Reality Gait Task

We calculated the percentage of time spent freezing and the footstep latency coefficient of variation for both the plank and normal walking conditions. A set of pairwise *t* tests were performed on the differences between the narrow and wide plank conditions (*P >* 0.05). Given that no differences were found between the wide and narrow planks across multiple FOG measures, we pooled the two plank conditions by calculating the sum of the narrow and wide plank measures for all sessions and calculated the average of the pooled plank measures across each participant. We performed pairwise *t* tests to compare the pooled plank (threatening) conditions to the normal walking (non‐threatening) conditions.

## Functional MRI Acquisition and Pre‐Processing

A General Electric 3T MRI (Boston, United States of America) was used to collect T_2_‐weighted echo‐planar functional images, acquired in sequential order with: repetition time = 3000 ms; echo‐time = 40 ms; flip‐angle = 90°; 40 axial slices covering the entire brain; interslice gap = 0.4 mm; field of view = 220 mm; and the raw voxel size = 3.9 × 3.9 × 4.0 mm. A high‐resolution 3D T_1_‐weighted anatomic image with voxel size = 0.4 × 0.4 × 0.9 mm was obtained for co‐registration with functional scans. Precautions were taken to control for the impact of head motion: all participants were instructed to keep their head motionless, and cushions were placed between participant's head and head coil to limit physical movement of head. Pre‐processing of images was performed using FMRIPREP (Supplementary Appendix S1 for further details).

### Region of Interest Selection

We selected a set of predefined regions of interest (ROIs) relating to motor, limbic, and cognitive networks based on previous work.^14^ We selected cortical ROIs from each of these groups from the 17‐network 400‐region Schaefer cortical parcellation^39^: the motor/supplementary motor area network, cognitive control network, and limbic networks. The subcortical regions chosen included bilaterally the nucleus accumbens, caudate, putamen, amygdala, and thalamus from the Harvard‐Oxford subcortical parcellation.^40^, ^41^, ^42^, ^43^ The periaqueductal gray was selected by mapping manual segmentation of voxels according to the following coordinates (MNI x: 0; y: −32; z: −8.5 plus 6.0 × 2.0 × 1.5 mm).^44^

## Dynamic Functional Connectivity Analysis

The pre‐processed and denoised functional data underwent time‐series extraction for the selected ROIs (Supplementary * for further details). To determine the time‐resolved dynamical functional connectivity between the selected 150 ROIs, we used the multiplication of temporal derivatives (MTD) approach.^45^ Using this approach, a region × region × time tensor was defined for each session. To determine the distribution of the connectivity nodes across the network, we calculated the participation coefficient^46^ using the Brain Connectivity Toolbox.^36^ The participation coefficient, B_T_, which quantifies the extent that a region connects across modules, was calculated for each time‐resolved dynamic functional connectivity matrix (Supplementary * for additional detail).

## Statistical Analysis

We fitted the time‐resolved connectivity values to a general linear model that included separate regressors for epochs of normal walking, plank walking and freezing, each of which were convolved with a canonical hemodynamic response function. For each session, we calculated the contrast between the dynamic functional connectivity parameter estimate for “plank walking‐normal walking”, after controlling for freezing events. We controlled for overt freezing events because we were explicitly interested in the neural mechanism that sets the stage for an individual to freeze. Finally, the average difference in pairwise connectivity was determined for each pair of regions. We controlled for multiple comparisons by running a non‐parametric permutation test, comparing the difference in between the plank and normal walking conditions for both dynamic functional connectivity and participation coefficient. The permutation test is a significance test that computes all possible values of the test statistic under all rearrangements of the observed data points by randomizing the condition labels (further description in the Supporting Data).^47^

Finally, we investigated the relationship between self‐reported anxiety measures and the difference in dynamic functional connectivity (cross‐talk between networks) between the plank and normal walking conditions. We calculated the Pearson's correlation between self‐reported anxiety and the difference between the dynamic functional connectivity between plank and normal walking conditions for each participant. Only significant correlations were reported that survived permutation testing (*P* < 0.05).

### Pupillometry

Fluctuations in pupil diameter were collected whilst participants performed the same VR gait task outside the scanner in the *off* state, because of the incompatibility of the Pupil Lab eye tracker and MRI scanner. The Pupil Lab software analyzed each frame produced by the eye outputs, and used sophisticated algorithms that detect contours of specific criterions; curvature continuity, and histogram pixel intensity to calculate the pupil diameter at each frame.^35^, ^48^, ^49^ The pupillometry analysis tracked pupil fluctuations of participants across the VR gait task, specifically focusing on the fluctuations in pupil diameter comparing differences between the normal walking and the plank condition. The pupil response for the two conditions were defined as the first 3 seconds after the onset of each task condition, baseline corrected using the average pupil response from 1 second before the onset (more information in the Supplementary *).

## Results

### VR Gait Task

We observed a significant increase in the percentage of time spent frozen during threatening compared to the non‐threatening condition (*P* = 1.86 × 10^−4^) (Fig. 1). Similar differences were observed for coefficient of variation of step‐time (*P* = 1.96 × 10^−5^), number of freezing events (*P* = 2.45 × 10^−6^), and the duration of freezing events (*P* = 6.88 × 10^−4^). We did not observe any effect of plank width on the different outcome measures of FOG.

## Dynamic Functional Connectivity

**FIG 2:**
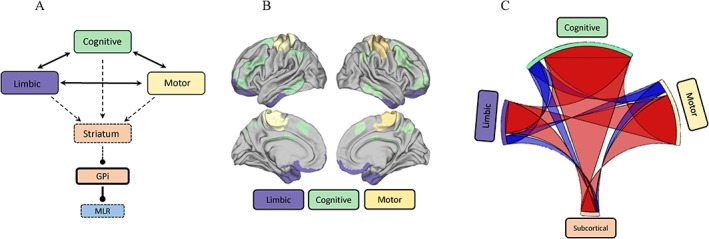
Dynamic functional connectivity analysis of threatening versus non‐threatening conditions. (**A**) Cross‐talk model depicted graphically, with cross‐talk visualized through connections between cognitive, motor and limbic networks; with corresponding influence on the striatum (Str), which inhibits the globus pallidus internus (GPi), and subsequently inhibits the mesencephalic locomotor region (MLR). (**B**) Cortical networks represented on brain figure, colors representative of colors on circular graph plot. (**C**) Circular graph plot of all the positive (red) and negative (blue) functional connectivity for 150 regions (bilaterally) in plank versus normal walking conditions, grouped into functional networks. A total of 830 significant pairs (significance calculated using permutation testing, *P* < 0.05). Functional connectivity was calculated using the multiplication of temporal derivatives. β values were calculated using a generalized linear model for plank (threatening), normal walking (non‐threatening), and freezing conditions. Line width in graph represents the weighted connections between networks, for example the larger width indicates more connections between regions in one network to the other network. The distribution of network across the circle plot relates to the ratio of individual nodes pertaining to the categorized network. [Color figure can be viewed at wileyonlinelibrary.com]

**FIG 3:**
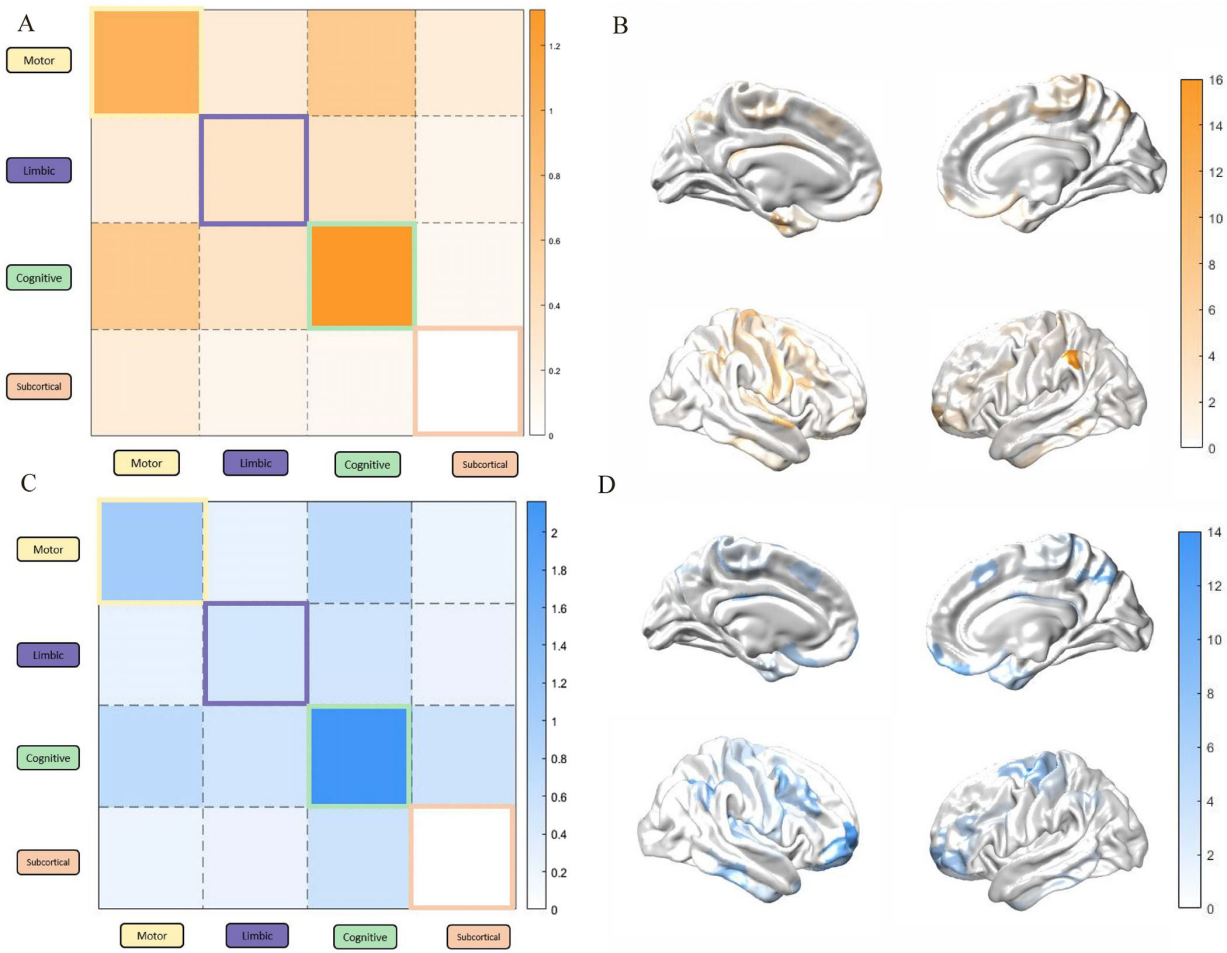
Difference in FOG severity relates to distributed coupling across the brain. (**A**) Summed percentage of edges within a network that is positively (orange) correlated with the change in percentage of time‐spent frozen (plank–normal). (**B**) Brain surface plots of the summed positive correlations across the cortex. (**C**) Summed percentage of edges within a network that is negatively (blue) correlated with the change in percentage of time‐spent frozen (plank–normal). (**D**) Brain surface plots of the summed negative correlations across the cortex. Pearson's correlations were performed between the positive coupling (β values) compared to the change in percentage of time‐spent frozen (plank–normal) (significance calculated using permutation testing, *P* < 0.05). Functional connectivity was calculated using the multiplication of temporal derivatives. Β values were calculated using a generalized linear model for plank (threatening), normal walking (non‐threatening). [Color figure can be viewed at wileyonlinelibrary.com]

We observed substantial increases in dynamic coupling within the brain during the threatening condition (Fig. 2). In total, there were 646 significant positive paired values and 184 negative paired values (*P* < 0.05 following a non‐parametric permutation test). The connectivity changes were distributed, with substantial increased connectivity between the motor and cognitive networks, and decreased connectivity within the limbic network (Supplementary Table S1). Coupling across multiple networks during the threatening condition was significantly positively correlated with multiple self‐reported anxiety measures (Supplementary Table S2). In addition, individual changes in the severity of freezing during the threatening condition (compared to normal walking) were related to increased cross‐talk between cognitive and motor networks, but decreased coupling within distinct regions of the cognitive network (Fig. 3). These results link abnormalities in functional connectivity to both anxiety and freezing (Table 1).

**TABLE 1:** Demographic, neuropsychological, clinical data

Variable	N = 26
Sex	19 M, 7F
Age, y	67.90 ± 6.6
Disease duration	11.60 ± 4.7
UPDRS‐III	37.40 ± 14.2
MMSE	27.20 ± 2.3
MOCA	24.64 ± 2.97
TMT‐A (s)	42.26 ± 19.3
TMT‐B (s)	118.9 ± 61.1
FOGQ3	2.43 ± 1.0
PAS – total	24.90 ± 15.9
PAS – persistent	6.93 ± 4.6
PAS – episodic	3.77 ± 3.0
HADS – total	9.97 ± 6.1
HADS – anxiety	4.97 ± 3.4
HADS – depression	5.00 ± 3.3
% Whole time spent frozen – normal	2.57 ± 2.2*
% Whole time spent frozen – plank	15.70 ± 11.4*
Mean foot‐step latency – normal (s)	0.518 ± 0.264
Mean foot‐step latency – plank (s)	0.849 ± 1.12
Total FOG events	16.40 ± 11.04
Mean duration of FOG event (s)	4.06 ± 5.57
Coefficient of variation without FOG	31.84 ± 15.60
Coefficient of variation with FOG	71.29 ± 39.64

Motor symptom severity was assessed using Part III of the MDS‐UPDRS. FOG was assessed both clinically and with questionnaires including the Freezing of Gait Questionnaire (FOGQ), and the Characterization of FOG questionnaire (CFOG). FOGQ3 refers to Freezing of Gait Questionnaire, Question 3 that specifically asks whether individual experiences freezing of gait. Cognition was assessed with the Montreal Cognitive Assessment (MoCA), the Mini‐Mental State Examination (MMSE), and the Trail Making Test (parts A and B). Affective disturbance was assessed using the Hospital Anxiety and Depression Scale (HADS) and Parkinson's Anxiety Scale (PAS).

* *P* < 0.05 statistically significant.

### Network Topology

**FIG 4:**
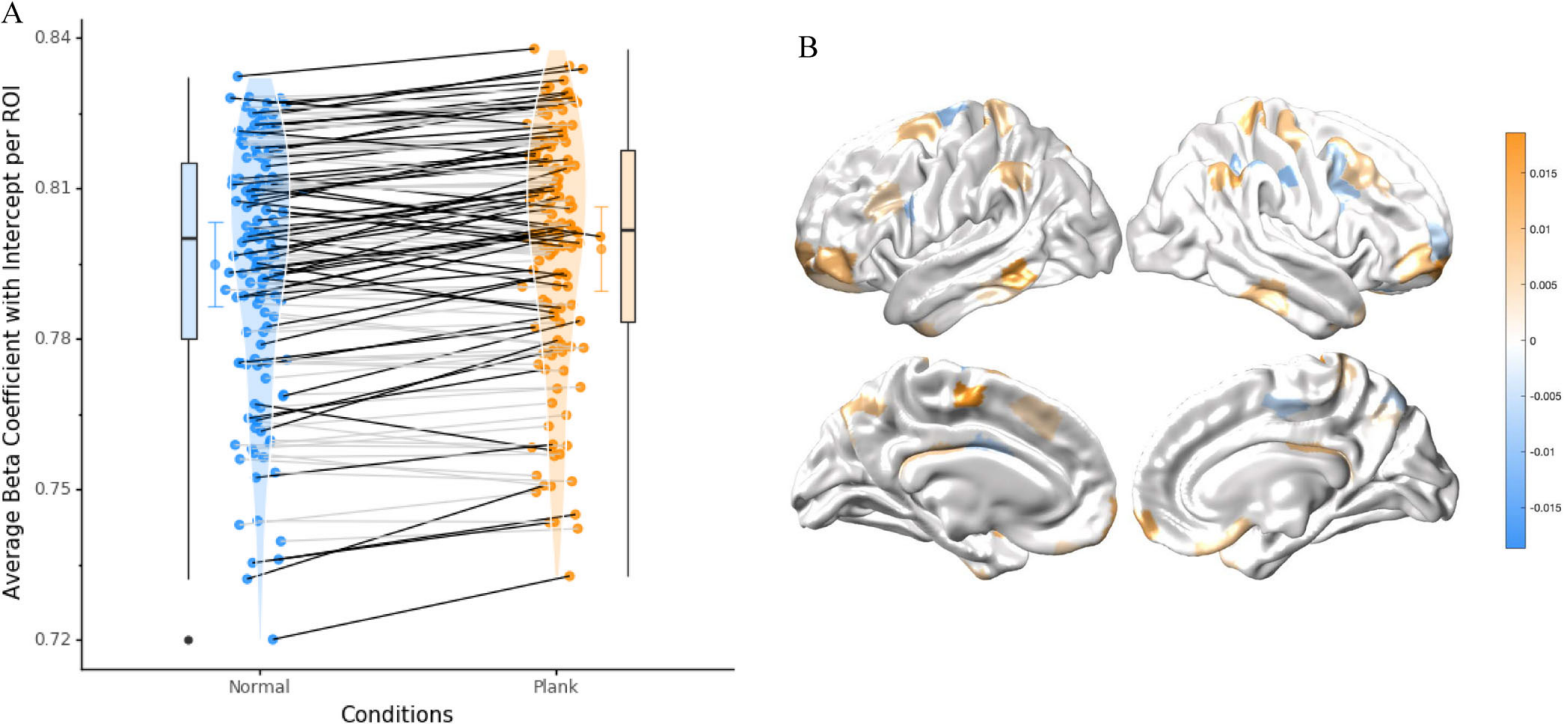
Increased regional integration during plank walking. (**A**) Mean β coefficient values including y‐intercept for each ROIs for threatening (plank) compared to non‐threatening (no plank aka normal) conditions, 64 regions of significance (indicated by black lines) calculated by permutation testing of 5000 random re‐clustering iterations across the two conditions (*P* < 0.05; Cohen's *d* effect size = 0.34). (**B**) Change in mean β coefficient values including for each ROIs for threatening (plank) compared to non‐threatening (no plank aka normal) conditions, plotted onto cortical surface, significance calculated by permutation testing of 5000 random re‐clustering iterations (*P* < 0.05). Participation coefficient was calculated using a generalized linear model of the participation coefficient values (clustering across network) and corresponding epochs of threatening and non‐threatening conditions, to obtain β coefficient values. [Color figure can be viewed at wileyonlinelibrary.com]

We have used graph theoretical approaches to summarize systems level features of brain network connectivity. We operationalized the notion of cross‐talk by estimating the extent to which individual regions of the brain “participate” in multiple communities, giving a participation coefficient value. There were 64 significant regions (*P* < 0.05, non‐parametric permutation testing) with increased participation coefficient values (*B*_*T*_) in the threatening versus non‐threatening condition distributed across the bilateral motor network, the limbic network, and the cognitive control network (Fig. 4).

### Pupillometry

**FIG 5:**
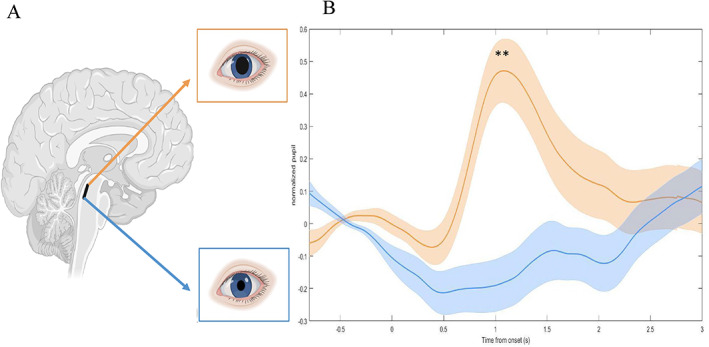
The average pupil dilation of all participants across all trials, for the threatening (orange) and non‐threatening (blue) conditions. (**A**) Visualization of relationship between LC pupil dilation between the normal (blue) and plank (orange) conditions. (**B**) Linear plots of the averaged normalized pupil response 3 seconds during the commencement of the conditions. The normal (blue) and plank (orange) conditions (events were pooled across narrow and wide conditions), with the standard deviation as the faded lines, significant difference observed for first 265 frames (2.12 seconds; *P* < 0.026 FDR; Cohen's *d* effect size = 1.90). A maximum peak between 1.09 and 1.96 second, indicated by **. [Color figure can be viewed at wileyonlinelibrary.com]

We observed significantly increased pupil dilation in the threatening condition for the first 265 frames (*P* < 0.026, FDR). An average maximal difference in the normalized pupil diameter between the threatening and non‐threatening condition was 0.70 (Fig. 5).

## Discussion

In this study, we tested the hypothesis that anxiety‐inducing contexts may lead to heightened noradrenergic tone, which sets the stage for freezing.^32^ By combining a validated anxiety‐inducing VR gait task^2^ with a task‐based dynamic functional connectivity analysis, we manipulated anxiety during the task through a set of virtual planks to determine the associated brain network topology. To further examine the neural mechanism underpinning the susceptibility to pathological cross‐talk, a subset of participants performed the same task outside of the scanner to obtain pupillometry. Using these multi‐modal approaches, our results refine leading models of FOG in PD, and implicate heightened ascending noradrenergic arousal as a potential augmenting factor in anxiety‐induced FOG.

### Anxiety‐Induced Dysfunctional Cross‐Talk

To our knowledge, this study was the first to use a task‐based fMRI analysis to determine contributions of anxiety to FOG. The comparison of the threatening and non‐threatening conditions revealed a wide‐spread increased coupling within and between the motor, limbic, and cognitive networks (Fig. 2). The cross‐talk across these networks during the threatening condition suggests that the networks were becoming more interconnected, perhaps through the biological mechanisms that instantiate anxiety in the brain, such as heightened sympathetic arousal.^23^, ^50^ We also observed increased dynamic cross‐talk within and across the subcortex during the threatening condition (Supplementary Table S1), highlighting a dysfunctional basal ganglia circuitry during the threatening condition. Typically, the basal ganglia work in segregated parallel processing pathways. However, in PD there is a loss of segregation across the striatum because of the functional demands exceeding the computational abilities of the dopamine‐depleted striatum.^51^ Cross‐talk from the cortical networks increases the processing demands on the basal ganglia and given the loss of segregation in the striatum in PD, it is unable to perform the parallel processing. The cross‐talk model explains the competition for neural resources across limbic, cognitive, and motor networks, which results in the overload of the striatum and basal ganglia circuit causing dysfunctional gait, such as freezing.^9^, ^52^ The cross‐talk model proposed that functional integration of normally segregated motor, cognitive, and limbic networks causes competing inputs onto the basal ganglia circuitry, leading to the globus pallidus internus/substantia nigra reticulata paroxysmally inhibiting the pedunculopontine nucleus, triggering a freezing episode.^9^ Hence, our study provides supporting evidence for the cross‐talk model because we found increased functional cross‐talk across competing and distributed networks in the brain.^9^

### Anxiety‐Induced Ascending Noradrenergic Arousal Drives Dysfunctional Integration

Our findings demonstrated increased integration across multiple cortical networks during the threatening condition, which likely occurs through anxiety‐related neural mechanisms. Using a similar analysis, a previous study found increased coupling across limbic, cognitive, and motor networks was associated with worse anxiety,^14^ suggesting that limbic interference could cause integration across cortical areas. Previous literature highlights that the threatening environment acts to engage “fight or flight” responses, likely through the noradrenergic arousal system,^53^, ^54^, ^55^ as evidenced in our findings (although pupil dilation has been linked to cognitive and attentional load).^56^ Furthermore, we established that the heightened sympathetic arousal because of the anxiety‐provoking walking environment^6^ may lead to an increased noradrenergic response throughout the brain, as evidenced by the pupillary dilation observed during the threatening condition. Notably, previous research has established a neural mechanism for driving network topology shifts, whereby increased noradrenergic input leads to integration across cortical networks.^24^, ^57^ In addition, previous computational modelling has highlighted a precise neural mechanism, by which an increase in noradrenergic response because of heightened sympathetic arousal, could integrate the brain—namely, through an increase in neural gain.^8^, ^58^, ^59^ The increase in noradrenaline raises the response gain of cortical regions across the network, facilitating competition between the motor and limbic networks that ultimately results in an inability to resolve the conflict and engage resources for motor control.^60^, ^61^, ^62^ This competition across cortical networks is further evidenced by our findings and links to previous literature.^10^, ^11^, ^14^ Together with the findings from the current study, we provide further evidence for increased cross‐talk because of elevated limbic load, which increases an individual's vulnerability toward interference and ultimately freezing episodes.

### Implications

Our findings demonstrate the noradrenergic arousal system undoubtedly plays a role in shaping the manifestation of anxiety‐induced FOG. However, the implications of our findings extend beyond FOG and reveal a missing piece of the puzzle to the pathophysiology of PD. Given the inherent complexity of PD and our current understanding of the contributions of the dopaminergic system to pathophysiology of PD, it is likely that other neuromodulatory systems (ie, cholinergic, serotonergic) are involved in the diversity of symptoms of PD.^63^, ^64^ It has previously been established that both acute and chronic anxiety plays a distinct role in PD.^65^ Other studies have established that anxiety worsens other symptoms of PD, such as bradykinesia^66^ and tremor.^30^ Furthermore, stress has also been evidenced to reduce the effects of dopaminergic medication.^31^ We propose that the ascending noradrenergic system during anxiety‐provoking contexts could result in overly integrated cortical brain states, which could then drive interference within the basal ganglia system resulting in manifestations of other motor symptoms of PD. Previous research has established that pharmacological inhibition of the ascending noradrenergic system and guided cognitive based relaxation can reduce motor symptoms,^67^, ^68^, ^69^ further establishing that noradrenergic arousal system could be driving anxiety‐induced motor symptoms of PD, and importantly providing potential novel therapeutic interventions.^69^ Whether through pathological inclusion, cell death, or non‐linear compensatory mechanisms, the noradrenergic arousal system undoubtedly plays a more important role in shaping the manifestation of symptoms in PD across motor, cognitive, and limbic domains, than has been previously appreciated.^70^

### Limitations

Given that we were unable to measure anxiety and pupillometry simultaneously during the fMRI‐task because of equipment constraints, we were unable to draw direct causal relationships. However, future research will examine pupillometry during the same anxiety‐inducing VR‐gait paradigm inside the MRI scanner. Hence, we have used a VR gait paradigm that has been validated to induce anxiety‐provoked freezing in both the VR and in real‐life environment.^2^ We acknowledge that a limitation is that within our paradigm there was no cognitive control, and cannot directly delineate the contributions of greater cognitive processing occurring during the threatening condition, which could also contribute to pupil dilation. However, both emotional and cognitive processing could be critical in contributing to the manifestation of freezing.^7^, ^11^, ^14^ More specifically, anxiety could interact with heightened cognitive processing in FOG.^71^ In future research, we intend to investigate this by using a previously validated dual‐tasking version of our VR‐gait paradigm in combination with the plank walking described here.^11^

## Conclusion

Overall, FOG is a complex phenomenon that encompasses cognitive, motor, and anxiety features. Limited research has determined the interactions between all these features, specifically the interaction between anxiety and FOG. Our results suggest that a deeper understanding of the pathophysiology of PD may be augmented through an appreciation of the complex inter‐relationships that characterize the ascending noradrenergic arousal system and its impact on functional neural networks.^30^ It should be noted that our findings reveal that anxiety may be driving a specific neural mechanism that predisposes an individual to experience anxiety‐related FOG, in which case treating anxiety in individuals with this subtype may be more beneficial compared to individuals who display other subtypes of FOG.^72^ Our work, therefore, provides a crucial link between neurobiological studies of the effects of anxiety and a real‐world clinical problem affecting a substantial proportion of individuals with PD. In conclusion, we have contributed by further advancing our understanding of FOG by proposing neural mechanisms, by which anxiety may be driving FOG manifestation. Further interrogation of these mechanisms will undoubtedly provide novel insights that will ultimately benefit the clinical management of this troubling neurodegenerative disorder.

## Author Roles

(1) Research project: A. Conception, B. Organization, C. Execution; (2) Statistical Analysis: A. Design, B. Execution, C. Review and Critique; (3) Manuscript: A. Writing of the First Draft, B. Review and Critique.

N.L.T.: 1A, 1B, 1C, 2A, 2B, 2C, 3A, 3B

G.W.: 2A, 2B, 2C, 3B

D.Q.: 2A, 2B, 2C

S.J.G.L.: 1A, 1B, 2A, 2C, 3B

J.M.S.: 1A, 1B, 2A, 2B, 2C, 3B

K.A.E.M.: 1A, 1B, 1C, 2A, 2B, 2C, 3B

## Financial Disclosures

This study was supported by Parkinson Canada (K.A.E.M.) and an Early Career Research Development Grant (K.A.E.M.) from the Brain and Mind Centre, University of Sydney. N.L.T. is supported by the Australian Government Research Training Program Scholarship (RTP). G.W. is supported by the Becas Chile Scholarship by Ministry of Science, Chile. J.M.S. is supported by a National Health and Medical Research Council (NHMRC) Project Grant (1156536) and University of Sydney Robinson Fellowship. S.J.G.L. is supported by NHMRC Leadership Fellowship (1195830). K.A.E.M. is supported by a New Investigator Award from Parkinson Canada, and a Discovery grant from the National Sciences and Engineering Research Council of Canada.

All the authors report no conflict of interests with this research study.

## Supporting information


**Appendix S1** Supporting Information

**Figure S1.** Freezing severity compared to summarized positive functional connectivity during plank versus normal walking conditions. Represents the summed positive functional connectivity values connectivity in plank versus normal walking conditions compared to the percentage of time‐spent frozen in the virtual‐reality gait paradigm (freezing severity). Functional connectivity was calculated using the multiplication of temporal derivatives. β values were calculated using a generalized linear model for plank (threatening), normal walking (non‐threatening), the positive β values were summed to produce a singular summary value of functional connectivity for each subject. The correlation was calculated using Pearson's correlation, and permutation testing for significance (*P* < 0.05). However, the summary measure used for the aforementioned correlation analysis (Supplementary Fig. S1) is problematic. Specifically, it is not clear whether the mean β‐weight across all pairs of regions is interpretable, because some subjects may have complex, network‐level features that are not expressed within individual edge weights that will be effectively washed out by this approach. Instead, we have opted to also include the correlation between each edge in turn (Supplementary *) and the total of percentage time frozen. To further understand the relationship between the brain functional connectivity and FOG in the virtual‐reality gait paradigm, we correlated the positive functional connectivity β values (calculated from the generalized linear model for plank vs. normal walking) with the total percentage of time‐spent frozen in the task.
**Figure S2.** Freezing severity correlates with increased coupling during plank versus normal walking conditions. Connectivity matrix plot, with dots representing the significant correlation values between the positive functional connectivity in plank versus normal walking conditions compared to the percentage of time‐spent frozen (freezing severity) (significance calculated using permutation testing, *P* < 0.05). The connectivity matrix represents the specific edges of significant correlation across the 150 brain regions that had increased coupling and were significantly correlated with the freezing severity. Functional connectivity was calculated using the multiplication of temporal derivatives. β values were calculated using a generalized linear model for plank (threatening), normal walking (non‐threatening). Positive (increased coupling) β values were correlated with percentage of time‐spent frozen.

**Table S1.** All the positive and negative functional connectivity for 150 regions (bilaterally), with specific subcortex connectivity in plank versus normal walking conditions, grouped into functional networks.
**Table S2.** The table depicts the specific pairs of nodes with increased ‘cross‐talk’ (coupling) during the plank condition compared to the normal walking condition that were significantly positively correlated with each measure of the Parkinson's Anxiety Scale.

## Data Availability

Data is available upon reasonable request to the authors. We have provided publicly available access to the codes used to conduct the analysis (see https://github.com/NatashaLTaylor/anxiety-gait-task-fMRI-in-FOG-PD).
