# Supplementary material for: The Contribution of Noradrenergic Activity to Anxiety‐Induced Freezing of Gait
Source: Mov Disord. 2022 Apr 5;37(7):1432–43. doi: 10.1002/mds.28999 (PMC9540856; doi:10.1002/mds.28999)
Supplement: Supplementary file 1 — Appendix S1 Supporting Information [file MDS-37-1432-s002.docx]

# **Supplementary Materials**

## **Clinical and Neuropsychological Assessment**

Motor symptom severity was assessed using Part III of the MDS-UPDRS. No participants reached MDS criteria for Parkinson’s Disease with Dementia. FOG was assessed both clinically and with questionnaires including the Freezing of Gait Questionnaire (FOGQ), and the Characterization of FOG questionnaire (CFOG). Cognition was assessed with the Montreal Cognitive Assessment (MoCA), the Mini-Mental State Examination (MMSE), and the Trail Making Test (Parts A and B). Affective disturbance was assessed using the Hospital Anxiety and Depression Scale (HADS) and Parkinson’s Anxiety Scale (PAS) (see Table 1). Participants with current psychiatric disorders were excluded from the study.

## **Virtual Reality Gait Task**

Participants lay supine inside the MRI scanner with a mirror mounted to the head coil. This allowed the participant to see a screen that was projecting the virtual gait task from the Dell laptop (Dell Technologies Inc., TX., US) outside the bore of the scanner. Participants were instructed to flex and extend their ankle so that they could alternatively tap pedals that were placed under their feet in the scanner. This alternative depression of the foot pedals (left-right-left…) allowed the participant to manoeuvre forward through the virtual environment and encoded binary inputs corresponding to left and right footsteps were recorded on the computer. The foot pedals were connected to a laptop by a long extension lead through universal serial bus (USB) connection and logged all event times and responses during the virtual-reality gait paradigm.^38^

The virtual environment consisted of a series of corridors (first-person view), that after turning a 90^o^ degree corner would reveal either a normal corridor or a corridor with a plank to cross. The plank condition contained two types; a narrow and a wide plank.^3^ The planks were suspended above a dark trench, under which there was no ground visible. Commencement of the task was signalled by a ‘WALK’ cue, written in large green letters across bottom of the screen, which was also synchronised to the MRI scanner. Participants were required to continue the task until signalled to ‘STOP’ written in large red letters, followed by the cue ‘END’, signifying completion of the task (upon completion of fMRI scan). Participants were familiarized with the task once prior to scanning, ensuring correct performance and understanding of the task. Participants performed 2-3 different runs of the virtual-reality gait paradigm. In each run, participants completed several blocks that were randomized to alternate between normal walking, narrow-plank and wide-plank conditions. The length of each condition was not specified because it was determined by the cadence (steps/min) of the participant. With each trial consisting of ~8(± 2) steps for normal walking condition, an estimate of average trial length was 4.06 sec. In the plank walking condition, on average an individual took ~16(± 5) steps, an estimate of the average trial length was 13.53 sec. However, this was variable given that it depended on the individuals cadence during that trial. Depending on the cadence of the participant, they could complete a varying number of conditions until 5 minutes of scanning was completed (on average 9 trials of each condition). Step length, width and velocity is pre-determined within the virtual-reality gait paradigm, and thus is outside of the participants’ control. Rather, the participants controlled the timing of the pedal depressions which execute a standard step. The foot pedals were configured to present ‘out of sequence’ steps if continuous ‘left-left, or right-right’ steps are taken or if participant does not press pedals, indicating participant is not performing gait paradigm correctly.

Two participants did not fully complete the task in the scanner due to the severity of freezing that was provoked during the plank condition (these sessions were removed from further analysis), as indicated by continuous ‘out of sequence’ recordings. One participant was removed due to incomplete clinical and psychological assessments.

The timing of each participants’ steps were recorded as the onset of depression (in milliseconds) of each foot pedal. The onset time of each condition event (i.e., ‘plank reached’) and the termination time of each event (i.e., ‘plank exited’) were also recorded. The duration of each condition event was calculated by determining the difference between onset time and termination time.

The percentage of time-spent frozen was calculated as the percentage of time spent freezing in either the plank or normal walking conditions, and the total percentage of time spent frozen in both conditions, over the total time spent walking in the virtual reality gait paradigm.

## **Functional MRI acquisition pre-processing**

Pre-processing of images was performed using FMRIPREP (version stable 1.5.8, a Nipype, 3,4 RRID:SCR_002502 based tool). T1-weighted anatomical images were intensity non-uniformity corrected using ‘N4BiasFieldCorrection’ (v2.1.0) and skull-stripped using ‘antsBrainExtraction’ (v2.1.0, using Oasis template^69^). Spatial normalization to the ICBM 152-Nonlinear Asymmetrical Template v2009c^70^ was performed using non-linear registration from ‘antsRegistration’ tool from ANTs (v2.1.0^71^) and the brain was extracted from the T1-weighted volume. Brain tissue was segmented using ‘fast’ into cerebrospinal-fluid (CSF), white matter (WM), and grey matter (GM) from the T1-weighted image.^72^ The functional data was slice-time corrected with ‘3dTshift’ from AFNI (v16.2.07^73^) and motion correct with ‘mcflirt’ from FSL (v5.0.9).^74^ The functional data was co-registrated to the corresponding T1-weighted image with six degrees of freedom and boundary-based registration using ‘flirt’ from FSL. The motion-corrected transformations (Bold-to-T1w and T1w-to-template [i.e., MNI space]) were concatenated and applied using ‘antsApplyTransformation’ (ANTs, v2.1.0). Physiological noise regressors were determined using CompCor.^71^ The principal components of CompCor were calculated for two variants; temporal and anatomical. Signal from the cortex were excluded using the brain mask. Six temporal CompCor components were defined by including only the top 5% most variable voxels. Six anatomical CompCor components were calculated using the intersection of subcortical mask and CSF and WM masks. Frame-wise displacement was calculated for each functional scan using Nipype.^75^

Denoising functional data involved using a toolbox ‘Denoiser’ (Tambini, Arielle and Gorgolewski, Krzysztof J., 2020; <https://github.com/arielletambini/denoiser>), which performs nuisance signal removal and temporal filtering of 4D functional data. The toolbox uses python (v3.) and imported packages to perform removal of nuisance signals at a voxel-wise level. Nuisance signals of the functional data that were computed in FMRIPrep. The chosen nuisance signals included the six head motions (transverse plane x,y,z, and rot plane x,y,z)and the corresponding first derivatives, anatomical CompCor, and temporal filtering. The nuisance signals were chosen as they were not correlated with each other and enabled us to regress out nuisance signals that contribute to significant noise in functional data without removing substantial signal that could be derived from true brain activity.^77,78^ The functional data was also band-pass filtered (0.01 – 0.1 Hz) to remove frequencies so as to control for noise from the MRI scanner and/or physiological noise.^79^

## **Dynamic Functional Connectivity Analysis**

We used the multiplication of temporal derivatives (MTD) approach, with a window size of 20TRs (i.e., 60sec). We estimated time-varying connectivity by first calculating the temporal derivative of each time series by performing the first-order temporal difference. For each node (*n*), with unique time points *(t*), a *t-1* vector of temporal derivatives was calculated and normalized by dividing the temporal derivative by the standard deviation of the temporal derivative. The MTD at each time point was calculated as each temporal derivative for each pair of nodes (*ij*) are multiplied, resulting in the value in each cells of the matrix reflecting the degree of functional coupling between the *i^th^* and *j^th^* nodes.^46^

### $\boldsymbol{MTD}_{\boldsymbol{ijt}}\boldsymbol{=}\frac{\boldsymbol{1}}{\boldsymbol{w}}\sum_{\boldsymbol{t}}^{\boldsymbol{t+w}} \frac{\boldsymbol{(}\boldsymbol{dt}_{\boldsymbol{it}}\boldsymbol{\times}\boldsymbol{dt}_{\boldsymbol{jt}}\boldsymbol{)}}{\boldsymbol{(}\boldsymbol{\sigma}_{\boldsymbol{dt}_{\boldsymbol{i}}}\boldsymbol{\times}\boldsymbol{\sigma}_{\boldsymbol{dt}_{\boldsymbol{j}}}\boldsymbol{)}}$ (1)

#### Equation 1- calculation of MTD, for each time point (t), pairwise interaction between region i and region j, dt is the first temporal derivative of the i^th^ and j^th^ time-series, and σ standard deviation of the temporal derivative, w is the window length of the simple moving average^46^.

$\boldsymbol{B}_{\boldsymbol{jt}}=\mathbf{1}- \sum_{\boldsymbol{s}=\mathbf{1}}^{\boldsymbol{n}_{\boldsymbol{M}}} \left( \frac{\boldsymbol{K}_{\boldsymbol{isT}}}{\boldsymbol{K}_{\boldsymbol{IT}}} \right)^{\mathbf{2}}$(2)

Equation 2- shows the participation coefficient, *B_jT_, K_isT_* is strength of positive connections of region *i* to regions in module *s* at specific time point *T, K_IT_* is sum of strengths of all positive connections of region *I* at time *T*. The participation coefficient is closer to 1, if the connections are distributed among all modules, and if participation coefficient is closer to 0, its connections are within its own module. Modules were clustered by the network assignment according to the Schaefer 17 Networks parcellation.

## **Statistical Permutation Testing**

The permutation test is a significance test that computes all possible values of the test statistic under all rearrangements of the observed data points by randomizing the condition labels. In this case, the permutation test generated random samples under the null hypothesis, with the randomized rearrangement of the condition labels of the plank and normal walking conditions. The null hypothesis was that there were no significant differences in dynamic functional connectivity (beta values) between the plank and normal walking conditions (i.e., condition labels of plank and normal walking are exchangeable). Hence, randomly permuting the condition labels of the observed results gives a new randomized result. Firstly, we calculated the difference in the means between the observed results (comparing the difference in COPEs between plank and normal walking), giving the test statistic. For each permutation, we calculated the difference between the means of the relabelled conditions (randomized reshuffling of the condition labels of plank and normal walking). The calculated differences are the distribution of possible differences under the null hypothesis that the condition labels are randomly assigned. The permutation p-value is computed as the proportion of the permutations (5,000 iterations), where the difference in the means was greater than or less than the test static of the 95^th^ percentile (p<0.05) of the new permuted differences.^48^

To determine the relationship between the difference in FOG severity and coupling ‘cross-talk’ across the brain we calculated the Pearson correlation (significance by permutation testing) between the positive beta values (‘coupling’) during the threatening condition (plank) vs non-threatening (normal walking) conditions, compared to the change in percentage of time-spent frozen in the virtual reality gait paradigm. We calculated the sum of the significantly correlated edges (‘coupling’ between nodes) and grouped them into their respective networks (motor, affective, cognitive, subcortex). Then, we determined the percentage of edges across the networks, by calculating the sum of the significantly correlated edges divided by the total possible sum of edges across a network (see Figure 3).

## **Pupillometry**

The participants (a subset of 25 subjects) were seated 2 metres in front of a 55-inch TV-screen (Samsung Smart TV, Samsung Electronics Co., LTD, Suwon, Korea) projecting from the Dell laptop (Dell Technologies Inc., TX., US) that displayed the virtual reality gait task (continuous walking for five minutes), and participants were instructed to depress their feet to alternately tap the foot pedals in the same manner as the fMRI-task. Fluctuations in pupil diameter of the eyes were collected using ‘Pupil Lab’ (Pupil Core product, Pupil Lab GmbH, Berlin., Germany) eye trackers at a sampling rate of 30 frames per second. The Pupil Lab glasses were worn by participants and connected to the Dell laptop through USB cable. These eye tracker functions using three cameras: the world camera (which provides insight into the participant’s field of vision – we used the world camera to time lock the virtual reality paradigm) and two eye cameras, which are individually directed towards the participant’s left and right eye. Manual adjustment of the eye cameras was performed to focus on the left and right pupil (pupil intensity focused – checked algorithm is registering participant’s pupil with calibration settings). After calibration, the Pupil Lab tracking accuracy was tested, with participants asked to look across different points on the screen. Artifacts, blinks, and outliers were removed from the analysis. To control for pupil affects from luminance in the task, we calculated luminance across the entire task, and determined that there was no statistical difference between the different conditions (corridors, narrow and wide planks) with luminance. We also controlled for luminance effects within the room, by performing the pupil task in the same room, with the same light on and limited window light. We detected points where the pupil derivative (rate of change) was higher or lower than 2.5 standard deviations from the mean, as this is a feature of abnormally increased or decreased pupil diameter. Blinks were corrected by sorting data for absence of pupil diameter and eliminating an extended margin of 100 msec to account for eye lids movement. A linear interpolation was performed to fill gaps as a result of eliminating data, and a lowpass second-order Butter-worth filter was applied at 2.5Hz. Z-scores were calculated for normalization of the data. The pupil diameter evoked response was then calculated using the average response across participants and was time-locked to the start of the virtual reality gait task.

**Supplementary Figures**

However, the summary measure used for the aforementioned correlation analysis (see Figure S1) is problematic. Specifically, it is not clear whether the mean beta-weight across all pairs of regions is interpretable, as some subjects may have complex, network-level features that are not expressed within individual edge weights that will be effectively washed out by this approach. Instead, we have opted to also include the correlation between each edge in turn (see Figure S2) and the total of percentage time frozen. To further understand the relationship between the brain functional connectivity and FOG in the virtual-reality gait paradigm, we correlated the positive functional connectivity beta values (calculated from the generalized linear model for plank vs normal walking) with the total percentage of time-spent frozen in the task.

## **Data availability**

Data is available upon reasonable request to the authors. We have provided publicly available access to the codes used to conduct the analysis (see <https://github.com/NatashaLTaylor/anxiety-gait-task-fMRI-in-FOG-PD>).
