# Supplementary material for: The Contribution of Noradrenergic Activity to Anxiety‐Induced Freezing of Gait
Source: Mov Disord. 2022 Apr 5;37(7):1432–43. doi: 10.1002/mds.28999 (PMC9540856; doi:10.1002/mds.28999)
Supplement: Supplementary file 2 — Figure S1. Freezing severity compared to summarized positive functional connectivity during plank versus normal walking conditions. Represents the summed positive functional connectivity values connectivity in plank versus normal walking conditions compared to the percentage of time‐spent frozen in the virtual‐reality gait paradigm (freezing severity). Functional connectivity was calculated using the multiplication of temporal derivatives. β values were calculated using a generalized linear model for plank (threatening), normal walking (non‐threatening), the positive β values were summed to produce a singular summary value of functional connectivity for each subject. The correlation was calculated using Pearson's correlation, and permutation testing for significance (P < 0.05). However, the summary measure used for the aforementioned correlation analysis (Supplementary Fig. S1) is problematic. Specifically, it is not clear whether the mean β‐weight across all pairs of regions is interpretable, because some subjects may have complex, network‐level features that are not expressed within individual edge weights that will be effectively washed out by this approach. Instead, we have opted to also include the correlation between each edge in turn (Supplementary *) and the total of percentage time frozen. To further understand the relationship between the brain functional connectivity and FOG in the virtual‐reality gait paradigm, we correlated the positive functional connectivity β values (calculated from the generalized linear model for plank vs. normal walking) with the total percentage of time‐spent frozen in the task. Figure S2. Freezing severity correlates with increased coupling during plank versus normal walking conditions. Connectivity matrix plot, with dots representing the significant correlation values between the positive functional connectivity in plank versus normal walking conditions compared to the percentage of time‐spent frozen (freezing severity) (signif [file MDS-37-1432-s003.pdf]

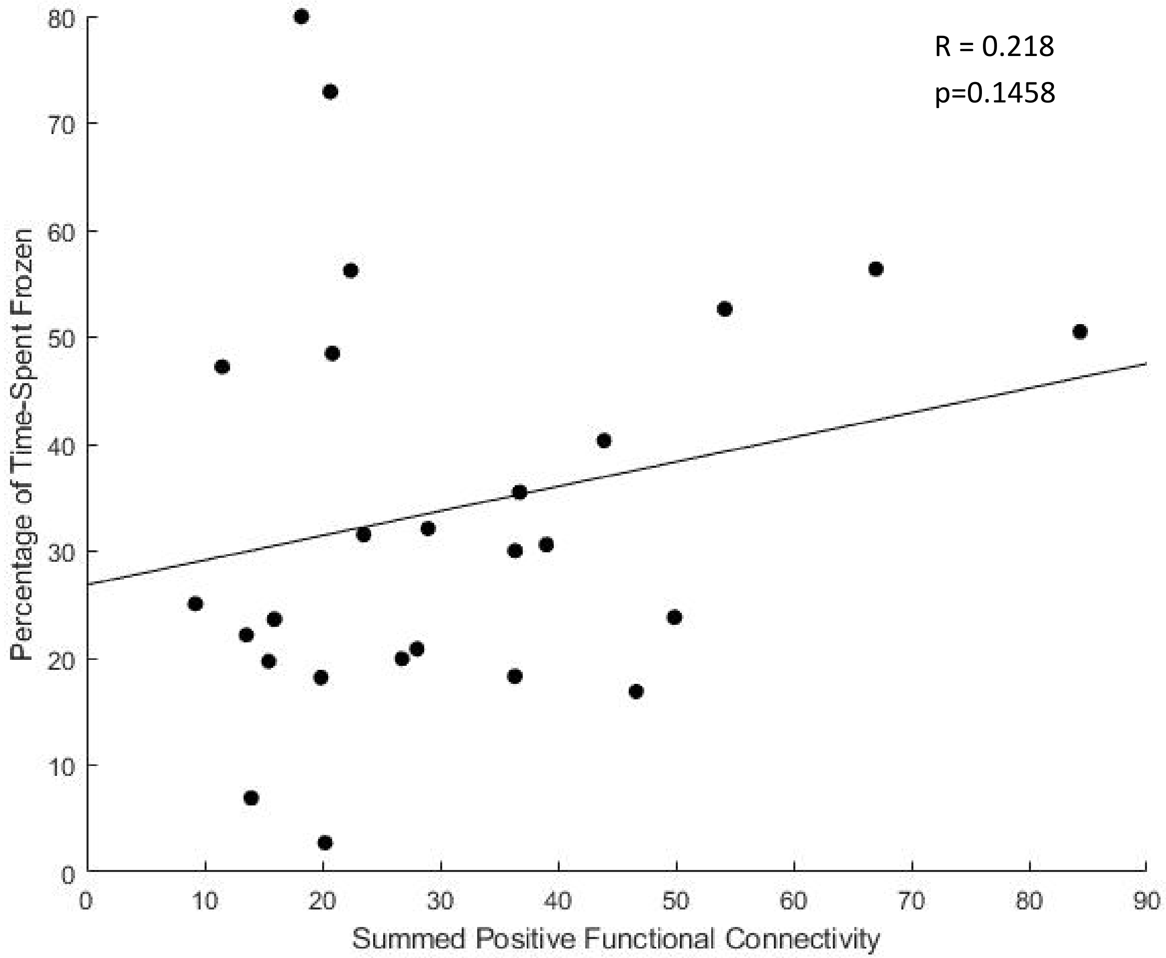

Figure S1: Freezing severity compared to summarised positive functional connectivity during plank versus normal walking conditions.

Represents the summed positive functional connectivity values connectivity in plank versus normal walking conditions compared to the percentage of time-spent frozen in the virtual-reality gait paradigm (freezing severity). Functional connectivity was calculated using the multiplication of temporal derivatives. Beta values were calculated using a generalized linear model for plank (threatening), normal walking (non-threatening), the positive beta values were summed to produce a singular summary value of functional connectivity for each subject. The correlation was calculated using Pearson's Correlation, and permutation testing for significance ($p<0.05$).

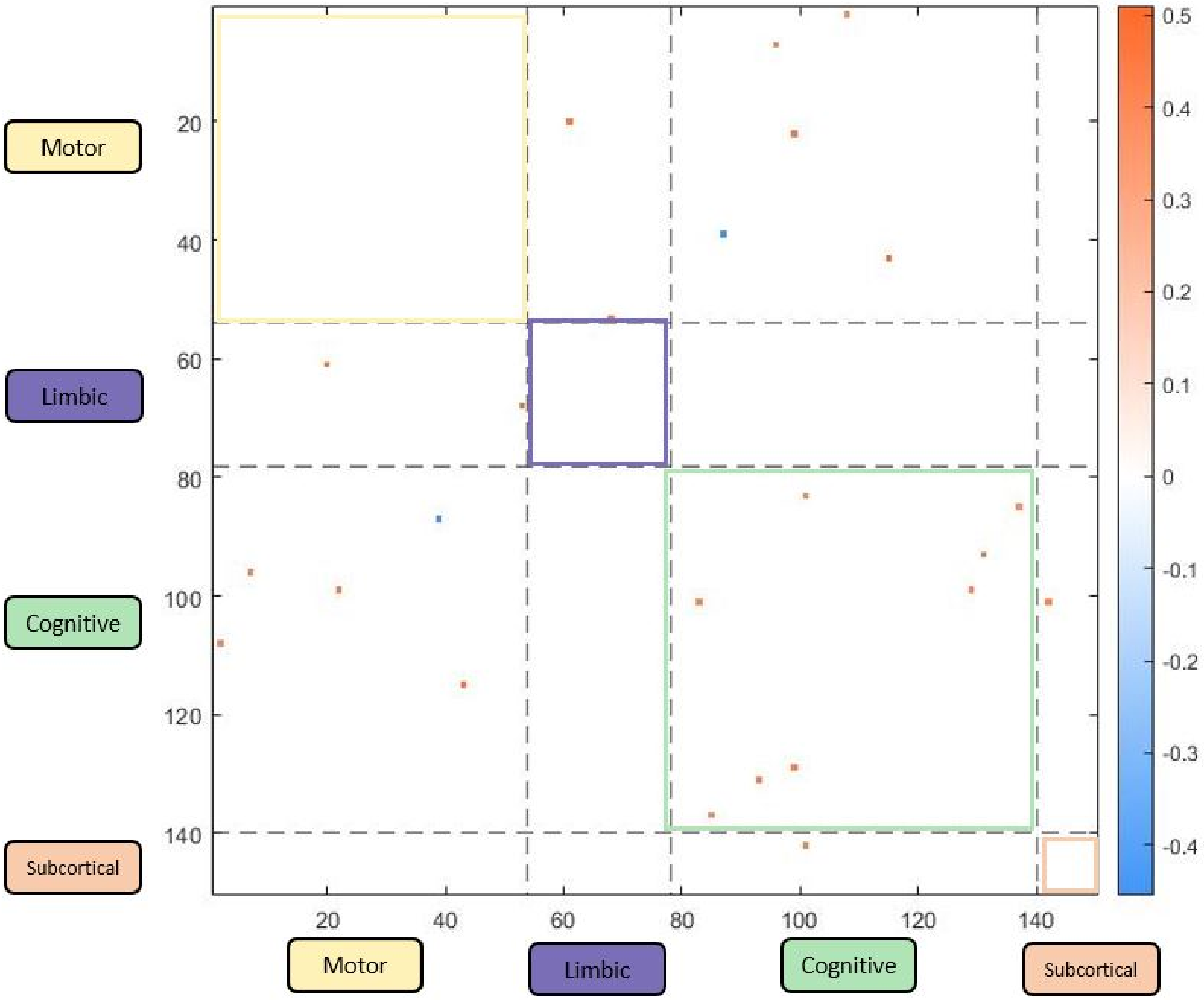

Figure S2: Freezing severity correlates with increased coupling during plank versus normal walking conditions.
Connectivity matrix plot, with dots representing the significant correlation values between the positive functional connectivity in plank versus normal walking conditions compared to the percentage of time-spent frozen (freezing severity) (significance calculated using permutation testing, $p<0.05$). The connectivity matrix represents the specific edges of significant correlation across the 150 brain regions that had increased coupling and were significantly correlated with the freezing severity. Functional connectivity was calculated using the multiplication of temporal derivatives. Beta values were calculated using a generalized linear model for plank (threatening), normal walking (non-threatening). Positive (increased coupling) beta values were correlated with percentage of time-spent frozen.
