# Supplementary material for: The Contribution of Noradrenergic Activity to Anxiety‐Induced Freezing of Gait
Source: Mov Disord. 2022 Apr 5;37(7):1432–43. doi: 10.1002/mds.28999 (PMC9540856; doi:10.1002/mds.28999)
Supplement: Supplementary file 3 — Table S1. All the positive and negative functional connectivity for 150 regions (bilaterally), with specific subcortex connectivity in plank versus normal walking conditions, grouped into functional networks. Table S2. The table depicts the specific pairs of nodes with increased ‘cross‐talk’ (coupling) during the plank condition compared to the normal walking condition that were significantly positively correlated with each measure of the Parkinson's Anxiety Scale. [file MDS-37-1432-s001.docx]

## **Supplementary Results Tables**

**Table 1: All the positive and negative functional connectivity for 150 regions (bilaterally), with specific subcortex connectivity in plank versus normal walking conditions, grouped into functional networks**.

| Positive Functional Connectivity | |
| --- | --- |
| Right Accumbens | Left somato-motor A (16) |
| Right Accumbens | Left limbic orbitofrontal cortex (5) (2x) |
| Right Accumbens | Left limbic temporal pole (6) |
| Right Caudate | Left somato-motor A (1) |
| Right Caudate | Left cognitive control A intraparietal sulcus (2) |
| Right Caudate | Left cognitive control B temporal (1) |
| Right Caudate | Left cognitive control B prefrontal cortex latero-ventral (3) |
| Right Putamen | Left somato-motor A (1) (2x) |
| Right Putamen | Left cognitive control A intraparietal sulcus (2) |
| Right Putamen | Left cognitive control C precuneus (2) |
| Right Amygdala | Left somato-motor A (3x) |
| Right Amygdala | Right cognitive control A intraparietal sulcus (1) |
| Right Thalamus | Right somato-motor A (2) (2x) |
| Left Accumbens | Right somato-motor A (12) |
| Left Accumbens | Right cognitive control A intraparietal sulcus (3) |
| Left Accumbens | Left Caudate |
| Left Caudate | Left somato-motor A (1) (4x) |
| Left Caudate | Left limbic orbitofrontal cortex (4) |
| Left Caudate | Left cognitive control B inferior parietal lobule (2) |
| Left Caudate | Right cognitive control B prefrontal cortex latero-dorsal (2) |
| Left Caudate | Left Accumbens |
| Left Putamen | Left limbic orbitofrontal cortex (2) (2 x) |
| Left Putamen | Left cognitive control A intraparietal sulcus (2) |
| Left Putamen | Right somato-motor A (12) |
| Left Putamen | Right limbic temporal pole (1) |
| Left Amygdala | Left somato-motor A (12) (3x) |
| Left Amygdala | Left cognitive control A prefrontal cortex lateral (2) |
| Left Thalamus | Left limbic temporal pole (6) |
| Left Thalamus | Left cognitive control B inferior parietal lobule (2) |

| Negative functional connectivity | |
| --- | --- |
| Left Accumbens | Left limbic temporal pole (2x) |
| Left Accumbens | Left cognitive control B inferior parietal lobule (1) |
| Left Accumbens | Right somato-motor A (7) |
| Left Caudate | Left somato-motor (3) (2x) |
| Left Caudate | Left cognitive control C cingulate (2) |
| Left Putamen | Right limbic orbitofrontal cortex (6) |
| Left Thalamus | Right cognitive control A prefrontal cortex lateral (2) |
| Right Caudate | Right cognitive control A intraparietal sulcus (4) |
| Right Caudate | Right cognitive control B prefrontal cortex latero-dorsal (4) |
| Right Putamen | Left cognitive control A prefrontal cortex lateral (2) |

830 significant pairs (significance calculated using permutation testing, p<0.05). Functional connectivity was calculated using the multiplication of temporal derivatives. Beta values were calculated using a generalized linear model for plank, normal walking and freezing conditions. Numbers indicate the specific region of interest in the Schaefer atlas.

**Table 2: The table depicts the specific pairs of nodes with increased ‘cross-talk’ (coupling) during the plank condition compared to the normal walking condition that were significantly positively correlated with each measure of the Parkinson’s Anxiety Scale.**

|  | Pairs of nodes with increased ‘cross-talk’ (coupling) across networks | |
| --- | --- | --- |
| Correlated across all measures of PAS | left somato-motor A | left somato-motor A |
|  | right somato-motor A | right cognitive control B temporal lobe |
|  | right somato-motor B | right cognitive control B inferior parietal lobule |
|  | right somato-motor B | right cognitive control B temporal lobe |
| Correlated with the PAS-total score | right somato-motor B | right caudate |
|  | right somato-motor B | left limbic orbitofrontal cortex |
|  | right cognitive control A intraparietal sulcus | left cognitive control C cingulate |
|  | right cognitive control B temporal | right somato-motor A |
| Correlated with PAS-episodic score | right somato-motor A | left cognitive control A intraparietal sulcus |
|  | left cognitive control A intraparietal sulcus | right cognitive control B prefrontal cortex lateroventral |
|  | left cognitive control A prefrontal cortex dorsal | right cognitive control C precuneus |
|  | right cognitive control intraparietal sulcus | left cognitive control C cingulate |
|  | right cognitive control B temporal lobe | right somato-motor A |
| Correlated with PAS-persistent score | right somato-motor A | left cognitive control A intraparietal sulcus |
|  | right cognitive control A cingulate | left cognitive control B prefrontal cortex latero-ventral |
